# The effect of MRI-based screening and selection on the prevalence of syringomyelia in the Dutch and Danish Cavalier King Charles Spaniels

**DOI:** 10.3389/fvets.2024.1326621

**Published:** 2024-01-29

**Authors:** Citlalli Limpens, Vivian T. M. Smits, Hille Fieten, Paul J. J. Mandigers

**Affiliations:** ^1^Expertise Centre of Genetics, Department of Clinical Sciences, Faculty of Veterinary Medicine, University of Utrecht, Utrecht, Netherlands; ^2^Evidensia Referral Hospital Arnhem, Arnhem, Netherlands

**Keywords:** breeding, welfare, Chiari, central canal dilatation, magnetic resonance imaging

## Abstract

**Introduction:**

Syringomyelia (SM) is a heritable disorder causing a fluid filled cavity (FFC) in the spinal cord with a reported overall prevalence of 39 to 46% in the Cavalier King Charles Spaniels (CKCS). Breeders started screening their CKCS with MRI in the Netherlands since 2004 and in Denmark since 2015. The goal of this study was to evaluate the effect of MRI-based selection in breeding on the prevalence of SM.

**Method:**

MRI scans of 2,125 purebred CKCS were available. SM was defined as having a visible FFC in the spinal cord. The prevalence of SM per year of birth was calculated, and a logistic regression was used to evaluate the affected status of offspring from affected versus unaffected parents and age category of the parent and study the combined effect of parental status and age-category to evaluate the effect on the affected status of the offspring.

**Results:**

The mean FFC in affected CKCS was 2.03 ± 1.47 mm and ranged from 0.5 to 9 mm (median of 1.5 mm). An age effect exists as older CKCS, which has a higher frequency of being affected compared with younger CKCS. There was no significant sex predilection for SM in this dataset. The mean prevalence of SM decreased slightly from 38% (2010–2014; 2.8 ± 1.3 years of age (mean ± sd); median 2.6 years) to 27% (2015–2019; 2.4 ± 1.2 years of age; median 2.1 years) in the screened population of CKCS (*p* = 4.3e-07). Breeding with two affected parents increased the odds ratio with 3.08 for producing affected offspring (95% CI 1.58–6.04) compared with breeding with unaffected parents.

**Discussion:**

MRI-based screening and selection against SM led to a minimal decrease in the prevalence of SM in the Dutch and Danish CKCS population. Breeding with dogs with SM significantly increases the risk of affected offspring. As the disorder is progressive with age, and based on the results of this study, MRI-based screening for all CKCS is recommended at an age of 3 years or older, and to reduce SM more effectively, CKCS affected with SM should not be used for breeding.

## Introduction

Several abnormalities that can lead to neurological signs have been identified in the Cavalier King Charles Spaniel (CKCS). Chiari-like malformation (CM also called canine Chiari) and syringomyelia (SM), first reported in 1997 (1), are most common (2). Other neurological abnormalities occasionally observed are ventriculomegaly (3–5), medullary kinking (6), atlanto-axial problems (6, 7), myoclonus (8), Medium-Chain Acyl-CoA Dehydrogenase Deficiency (9), and episodic falling (10, 11). The last frequently observed abnormality is the so-called glue ear (mucous in the tympanic bullae) and, by some authors, described as primary secretory otitis media (PSOM) (12). Although it is not a normal finding, it is unclear whether it has clinical significance.

Syringomyelia is defined as the presence of a cavity in the spinal cord (syrinx). In human medicine, if the central canal is slightly dilated in a localized region, it is called hydromyelia (13), but this term is rarely used in veterinary medicine. In veterinary medicine, syringomyelia (SM) has been defined as a central canal dilation of more than 2 mm. If it is less, the term central canal dilatation (CCD) is used (14). A better description is to name it a fluid-filled cavity/cavities (FFC) in the spinal cord as the ependyma of the central canal is disrupted after minimal dilatation (15). SM may also arise because of, for instance, intramedullary tumor, arachnoid adhesion, or a hydrocephalus (13, 14). In the CKCS, SM occurs without these disorders, and it is believed that in this breed, SM results from an abnormal CSF flow either related to hindbrain overcrowding and/or the influence of the systolic/diastolic pulse and CSF pulse on the subarachnoid spaces (5, 14, 16–19). Within the CKCS, the fluid-filled cavity/cavities/SM is often progressive (20, 21) and is therefore, in this breed, observed as a pathological disorder. Both CM and SM have, in the CKCS, a very high prevalence. In 2011, Parker et al. (20) reported an increasing prevalence of 20% for SM for dogs up to 1 year of age that rose up to 70% for dogs ≥6 years of age. The overall prevalence for the complete group of 555, originating from the United Kingdom and the Netherlands, CKCS was 46%. Moreover, in 2017, our group reported a prevalence of 99% for CM and an overall prevalence of 39% for SM in the examined group of 1,020, mainly Dutch, CKCS (21). The exact relation between these two disorders is, up to now, still not completely understood.

SM is highly heritable (heritability grade of 0.31–0.37), indicating that selection to decrease the prevalence of the disorder is possible (21, 22). In 2018, a candidate genetic locus associated with syrinx transverse diameter was identified, but the causal mutations have not been identified yet, and selection based on this locus has not been used for selective breeding strategies (23).

Breeders who want to reduce the number of SM-affected CKCS without making an MRI cannot rely on the clinical signs as only a limited number of CKCS, affected by CM or SM, show clinical signs (20, 21). Of interest, recent studies suggest that a more brachycephalic appearance of CKCS could lead to a higher incidence of CM and/or SM (24, 25), and for this reason, facial recognition software is developed and evaluated but is not yet reliable enough to be widely used to screen the breeding stock (26). Computed tomography (CT) is currently not a comparable alternative to MRI as it is considered inferior to MRI (27).

Hence, the only reliable tool for breed selection against CM and SM in CKCS is, up to now, MRI.

The fact that SM is a late onset disease makes the use of a young dog risky. A CKCS can be unaffected at a young age but affected at an older age (20, 21). CM/SM is not unique for the CKCS and has been reported to occur in several other brachycephalic toy-breeds, such as Chihuahua (28), Griffon (29), Affenpinscher (30), and Pomeranian (31).

As of 2004, breeders in the Netherlands began voluntary, and from 2011 mandatory (imposed by the Dutch Government and Kennel Club), screening of CKCS breeding stock with MRI to reduce the prevalence of CM and SM. In Denmark, the breeders started scanning on a voluntary basis in 2015, and as of this year, they are obliged to scan their breeding stock. When the breeders started scanning, they followed the recommendations published in 2007 (32), and as of 2012, a new scheme was introduced based on the outcome of screening of Dutch and United Kingdom CKCS (33). This scheme is now called the British Veterinary Association (BVA)/Kennel Club (KC) scheme.^1^ The dogs are also subdivided into three age groups. Age group ‘a’ means a dog older than 5 years of age, b means 3 to 5 years of age, and c means 1 to 3 years of age. CM is graded as 0 (no Chiari malformation) grade 1 (cerebellum indented (not round), cerebrospinal fluid (CSF) still visible between the cerebellum and brainstem), and grade 2 (cerebellum impacted into or herniated through the foramen magnum). SM is graded as SM0 which means no dilatation, SM1 up to 2 mm or less, SM2 a CCD of more than 2 mm.

Using this classification, breed recommendations were formulated allowing over 36 possible combinations of unaffected but also affected CKCS (33). Some breeders only use unaffected CKCS, but some breeders also use affected CKCS, which might limit the effect of selection against SM.

The goal of this study was to evaluate the effect of MRI-based selection in breeding on the prevalence of SM in the screened population of CKCS in Denmark and the Netherlands over the last two decades.

## Materials and methods

### Dogs

All included CKCS are purebred CKCS registered in the Netherlands, and a smaller number of CKCS registered in Denmark, Belgium, and Germany. The pedigree data were supplied by the Cavaliers for Life foundation, checked, and combined with information from the Dutch Kennel Club and the Danish Kennel Club. A CKCS was only included if the dog was screened with an MRI scan for breeding purposes. Moreover, a CKCS was evaluated as offspring if the dog had at least one parent that was screened by MRI.

### Pedigree data and quality control

Data analysis was performed in R^2^ and Excel.^3^ CKCS with incomplete records were removed. CKCS with double entry but different information (double registration number entry but for instance different parental or color information) were checked in the available Kennel Club dataset, and only the correct entry was kept. CKCS with no Kennel Club data available were removed from the dataset.

### MRI scan and classification of SM

For this study, all MRI scans of CKCS, born between 1998 and 2021, were used. When multiple scans were available from one dog, only the scan obtained at their highest age was used. All scans were made according to the guidelines of the BVA/KC, including transverse and sagittal T1-weighted and T2-weighted images of the head and the spinal cord up to at least C4. These MRI scans were submitted to the last author (PJJM) with an informed consent of the owner. MRI scans were received, in majority, from four different veterinary MRI centers: (1) Department of Clinical Sciences, Utrecht University, The Netherlands, (2) IVC Evidensia Referral Hospital Arnhem, The Netherlands, (3) Dierenkliniek den Heuvel, Best, The Netherlands, and (4) MR-Scanner.dk, Horsens, Denmark. The four MRI centers used either a low-field MRI scanner of 0.4 Tesla (Best) or a high field scanner of 1.5 Tesla (Utrecht, Arnhem, and Horsens). A small number of MRI scans, made before 2010, came from different MRI centers as published earlier (21). All MR images were re-evaluated using the Osirix DICOM viewer graphical software (34) by the last author (PJJM), an ECVN-board certified diplomat. All scans were evaluated using the BVA/KC scheme with one important adaptation: the presence of SM was established by the measurement of the exact width of the largest fluid-filled cavity in mm on the transverse T1-weighted images as differentiation between true syrinx margins and spinal cord edema surrounding the syrinx is more difficult on T2W than T1W images (35). The scans were further subdivided into three BVA/KC scheme age groups (a: older than 5 years of age, b: 3 to 5 years of age, and c: 1 to 3 years of age). Additional information included sex, date of birth, color, and age of the dog at scanning. The presence of CM and glue ear was reported but not further analyzed in relation to SM in the current study. In this study, SM unaffected CKCS were defined as those with a CCD of 0 mm, while those with a visible fluid-filled cavity (FFC) (> 0 mm), on at least two transverse images, were considered affected with SM.

### Exclusion criteria

MRI scans were excluded if the scan was not obtained according to the official guidelines and if a microchip artifact was present, preventing a proper evaluation of the spinal cord. CKCS for which parental information was unclear or unknown were removed from the dataset and CKCS with repeated ID entries when the IDs did not match the information available in the Kennel Club dataset. Furthermore, CKCS were removed which had unknown parental data, no registered family members, double registration/chip numbers, or a registration number that did not match the dataset of the Kennel club.

### Statistics

All statistical analyses were performed using R version 4.1.2 (2021-11-01). Descriptive statistics were calculated based on the percentage of SM-affected dogs per age category. Mean, standard deviation, range, and median were calculated for the syrinx diameter.

Logistic regression with ‘affected’ as dependent variable and sex and scan-age category of the CKCS as independent variables was performed to investigate the effect of age on the probability of being diagnosed with SM in the total dataset. The prevalence of SM was calculated by dividing the number of affected CKCS by the number of screened CKCS per year of birth.

Univariable and multivariable logistic regression models were used to predict the odds ratio for the offspring of being affected. Independent variables that were analyzed included: age of the offspring at the time of MRI scan (in years), sex of the offspring, SM-affected status of the combination of the parents (both free (reference category), 1 affected parent, 2 affected parents), and combination of the SM-scan age category of the parents (aa (ref category), ab, ac, bb, cb, cc). In the multivariable logistic regression, the model of best fit was determined using Akaike’s information criterion. Odds ratio, confidence intervals, and value of ps were reported.

## Results

### Description of the population

In total, 2,354 CKCS were available with MRI results. All included CKCS were registered in the Netherlands, Belgium, Germany, and Denmark but based on the pedigree analysis of the included CKCS, the ancestors of these CKCS came from the United Kingdom, the Netherlands, Belgium, France, Denmark, and Germany. After quality control, 229 CKCS were excluded. Hence, 2,125 dogs were available for prevalence analysis and 2065 CKCS (546 males, 1,491 females, and 28 animals with unknown gender) were available for pedigree analysis. The 60 dogs that were included for epidemiological analysis but not pedigree analysis had unknown recorded parents, or parentage was not confirmed via pedigree records. In total, 340 individuals had both maternal and paternal MRI information, and these trios were used for the odds ratio and parental effect calculations.

### MRI scan result SM from 2,125 CKCS

MRI scans from 2,125 CKCS were assessed. The prevalence of SM in the total dataset was 38.3% (815/2125). Affected CKCS had an FFC of at least 0.5 mm. The mean FFC in affected CKCS was 2.03 ± 1.47 mm and ranged from 0.5 to 9 mm (median of 1.5 mm). Of the affected CKCS, 511 CKCS would have been graded as SM1 (62.7%) and 304 as SM2 (37.3%), according to the BVA/KC scheme. Table 1 shows the number and percentage of affected dogs in the different BVA/KC age categories. The majority of the dogs were scanned in age-group c (1–3 years of age) *n* = 1,161 (54,6%), followed by age-group b (3–5 years of age) *n* = 778 (36,6%) and age group a (>5 years of age) *n* = 186 (8,7%).

**Table 1:** Overview of SM status per age group at MRI scan in the total dataset.

Age at MRI scan	Affected (% affected per age group)	Total
a (> 5 years)	95 (51,0%)	186
b (3–5 years)	390 (50,1%)	778
c (1–3 years)	330 (28,4%)	1,161
Total	815	2,125

In the total dataset, sex did not significantly influence the odds of being affected with SM. The age at which the MRI scan for the diagnosis was made significantly influenced the odds of being diagnosed with SM. Compared with a scan made at 1–3 years of age, the odds were, respectively, 1.88 (age group b) and 2.63 times (age group a) higher to be diagnosed with SM (Table 2).

**Table 2:** Effect of sex and age category on the affected status.

Total dataset of 2,125 dogs		
	Univariable analysis	
	Odds ratio (95% CI)	*p*-Value
Sex male (ref = female)	0.97 (0.78–1.2)	0.79
Age category (ref = c:1–3 years)	b (3–5 years): 1.88 (1.54–2.29) a (>5 years): 2.63 (1.92–3.60)	b: 4.27e-10 a: 1.71e-09

A logistic regression is used with the affected status as dependent variable and sex and age category as independent variables. Female sex was used as the reference category and age-category c (1–3 years) was used as the reference category.

### MRI scan result regarding CM and PSOM in the total dataset

CM grading was in four dogs scored as grade 0 (0,2%), 111 grade 1 (5,2%), and 2010 CKCS as grade 2 (94,6%). A glue ear, was observed in 20% of all right tympanic bullae, in 40% of all left tympanic bullae and in 11% of all CKCSs both tympanic bullae were affected.

### Prevalence of SM in 2125 CKCS screened with MRI between 1998 and 2021

**Figure 1:**
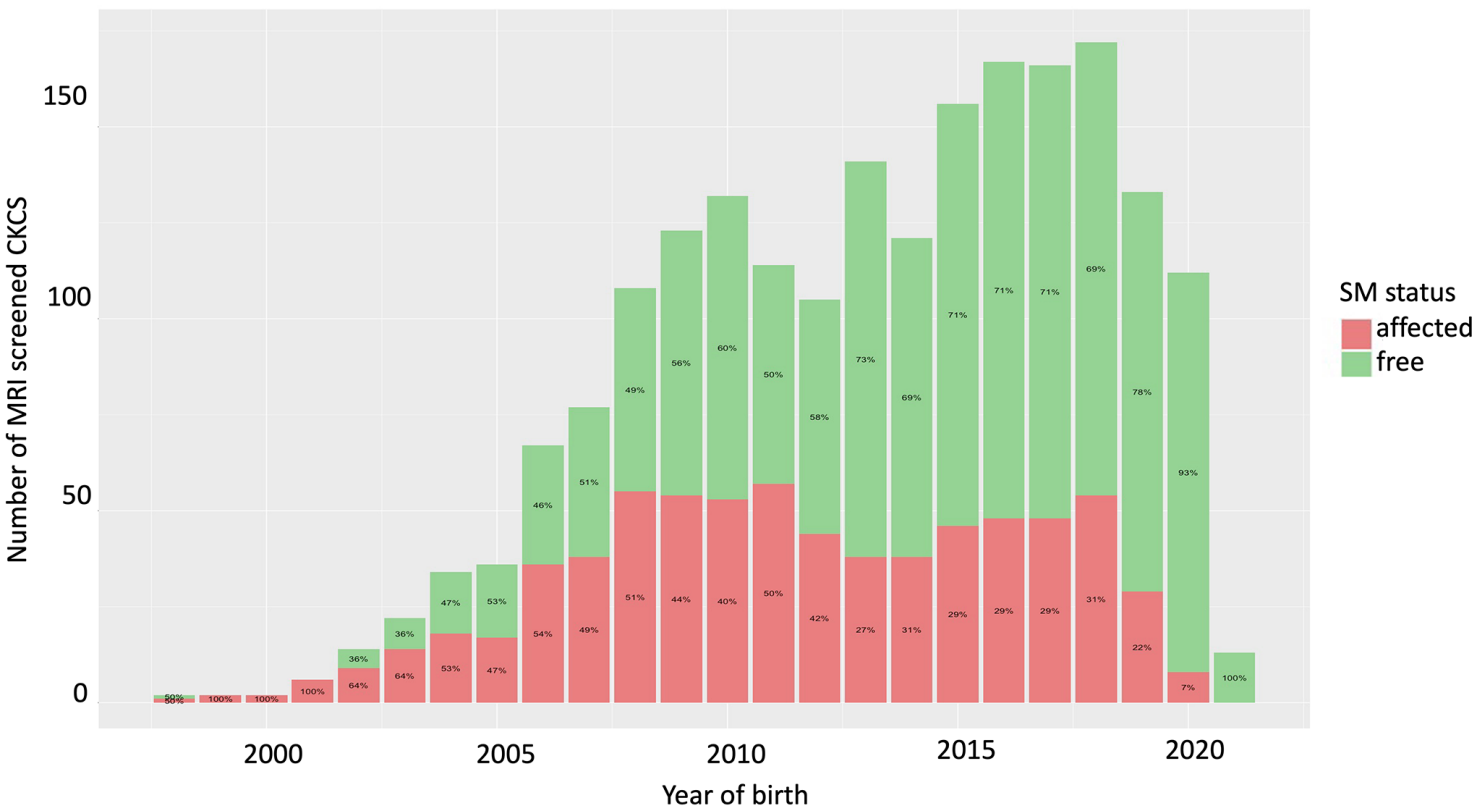
Number of CKCS that were screened using MRI from 1998 to 2021. Over time the number of unaffected CKCS increased and the ratio unaffected - affected CKCS decreased.

The total number of CKCS that was screened in the Netherlands and Denmark for birthyear and the SM status of the CKCSs are shown in Figure 1. The prevalence of SM decreased after the start of MRI-based screening, with the last generation (born between 2015 and 2019) having a mean prevalence of 27% (2.4 ± 1.2 years of age (mean age ± sd); median 2.1 years of age), while the generation before (2010–2014) had a mean prevalence of 38% (2.8 ± 1.3 (mean ± sd); median 2.6 years of age). Using the group born between 2015 and 2019 as the reference group, the difference between these two groups is statistically significant (logistic regression; *p* = 4.3e-07). The percentage for the different age groups for the scanned CKCS born between 2015 and 2019 was 60.9% for age group c, 34.6% for age group b, and 4.5% for age group a. The percentage for the different age groups for the scanned CKCS born between 2010 and 2014 was 69.2% for age group c, 24.5% for age group b, and 6.3% for age group a. This difference is not statistically different (chi-Square, df (9), *p* = 0.213).

### Percentage of SM-affected offspring and parent’s status

From 210 bitches and 95 sires that were scanned for breeding purposes, at least one offspring was scanned as well. Table 3 shows the results for the 210 bitches and Table 4 shows the results for the 95 sires. The logistic regression analysis is shown in Tables 5, 6. Notably, the majority of bitches (47.1%) and sires (53.6%) used for breeding had their SM status established in age category c (1–3 years of age).

**Table 3:** Percentage of affected offspring from 210 bitches.

Bitches	MRI scan age group	*n*	Offspring of the bitches			Percentage offspring SM affected
			Total (*n*)	Free (*n*)	Affected (*n*)	
SM free	a (>5 years)	16	33	23	10	30.3%
	b (3–5 years)	42	76	63	13	17.1%
	c (1–3 years)	60	143	98	45	31.5%
SM affected	a (>5 years)	18	28	16	12	50.0%
	b (3–5 years)	35	46	25	21	60.0%
	c (1–3 years)	39	64	40	24	68.6%

The total number of scanned offspring per bitch is shown. For each age group for the MRI scan, the number of unaffected and affected offspring was calculated. The last column shows the percentage of affected offspring for each category. The logistic regression analysis is shown in Table 5.

**Table 4:** Percentage of affected offspring from 95 sires.

Sires	MRI scan Age group	*n*	Offspring of the sires			Percentage offspring SM affected
			Total (*n*)	Unaffected (*n*)	Affected (*n*)	
SM free	a (>5 years)	15	85	62	23	27.1%
	b (3–5 years)	16	48	31	17	35.4%
	c (1–3 years)	31	113	82	31	27.4%
SM affected	a (>5 years)	8	29	19	10	34.5%
	b (3–5 years)	5	8	3	5	62.5%
	c (1–3 years)	20	76	45	31	40.8%

The total number of scanned offspring per sire is shown. For each age group for the MRI scan, the number of unaffected and affected offspring was calculated. The last column shows the percentage of affected offspring for each category. The logistic regression analysis is shown in Table 6.

**Table 5:** Logistic regression analysis of the offspring of the bitches.

Logistic regression analysis of offspring of the bitches				
	Univariable analysis		Multivariable model (model of best fit)	
	Odds ratio (95% CI)	*p*-Value	Odds ratio (95% CI)	value of p
Sex of offspring male (Ref = female)	1.18 (0.68–2.02)	0.541		
Age of offspring (in years)	1.44 (1.83–1.79)	0.00047	1.45 (1.17–1.79)	0.0007
SM status of the bitch affected (Ref = unaffected)	1.93 (1.27–3)	0.0025	1.89 (1.23–2.9)	0.003
Age-category MRI scan of the bitch (ref a: > 5 years)	b (3–5 years): 0.58 (0.31–1.09) c (1–3 years): 0.75 (0.42–1.34)	b: 0.08 c: 0.32		

**Table 6:** Logistic regression analysis of the offspring of the sires.

Logistic regression analysis of offspring of the sires				
	Univariable analysis		Multivariate analysis	
	Odds ratio (95% CI)	*p*-Value	Odds ratio (95% CI)	*p*-Value
Sex of offspring male (Ref = female)	1.43 (0.8–2.51)	0.218		
Age of offspring (in years)	1.49 (1.19–1.89)	0.0005	1.49 (1.19–187)	0.0006
SM status of the sire: affected (Ref = unaffected)	1.69 (1.06–2.69)	0.02	1.67 (1.04–2.68)	0.03
Age-category MRI scan of the sire (ref a: > 5 years)	b (3–5 years): 1.56 (0.79–3.06) c (1–3 years): 1.01 (0.6–1.71)	b: 0.2 c: 0.959		

The odds ratio for producing affected offspring when breeding a bitch affected with SM compared with breeding from an unaffected bitch is 1.89 (1.23–2.9). This odds ratio is corrected for the age of the offspring at the moment that the scan of the offspring was made. The odds ratio for producing affected offspring when breeding a sire affected with SM compared with breeding from an unaffected sire was 1.67 (1.04–2.68). The age category at which the scan of either the father or the mother was made was not significantly related to the affected status of the offspring in this analysis.

### SM status of 340 offspring with MRI scan results of both parents

Among the 340 dogs studied, the scan results were available for both parents, and Table 7 displays the outcomes regarding the percentage and number of SM-affected offspring for each potential parental combination. Out of these 340 dogs, 154 were descendants of two SM-unaffected parents (45.2%), while 50 had two SM-affected parents (14.7%). A total of 136 combinations involved one SM-unaffected and one SM-affected parent (40%). Within this dataset, the most frequently utilized combination featured two SM-unaffected parents from ‘age group c’ Cavalier King Charles Spaniels (n = 45 = 13.2%), followed by parents from ‘age group c and age group b’ CKCS (n = 44 = 12.9%). The most common combination involving one SM-affected and one SM-unaffected parent was ‘two age group c’ parents (n = 41 = 12%). Notably, the risk of producing affected offspring is significantly lower when using only unaffected parents compared with utilizing affected parents. The findings of a logistic regression analysis are presented in Table 8. The age at which the offspring undergoes scanning exhibits a significant association with diagnosis of SM. Specifically, an increase in age by 1 year corresponds to an increase in the odds ratio of 1.45 (CI 1.15–1.85) in the model of best fit. Parental status is also a significant factor in the best-fit model, with having two affected parents increasing the odds of being affected with SM by 3.08 (95% CI: 1.58–6.04). However, the combination of age categories of the parents does not show significance in the univariable analysis and is therefore excluded from the model of best fit.

**Table 7:** SM results for 340 offspring’s in different combinations of parents.

	Males					
Females	a (>5 years) SM free	b (3–5 years) SM free	c (1–3 years) SM free	a (>5 years) SM affected	b 3–5 years SM affected	c 1–3 years SM affected
a (>5 years) SM free	25.00% (*n* = 4)	20.00% (*n* = 5)	40.00% (*n* = 10)	0.00% (*n* = 1)	NA	50.00% (*n* = 2)
b (3–5 years) SM free	22.73% (*n* = 22)	0.00% (*n* = 7)	20.83% (*n* = 24)	0.00% (*n* = 4)	NA	18.18% (*n* = 11)
c (1–3 years) SM free	29.41% (*n* = 17)	45.00% (*n* = 20)	20.00% (*n* = 45)	20.00% (*n* = 5)	50.00% (*n* = 2)	29.63% (*n* = 27)
a (>5 years) SM affected	22.22% (*n* = 9)	100.00% (*n* = 1)	25.00% (*n* = 4)	71.43% (*n* = 7)	100.00% (*n* = 1)	33.33% (*n* = 6)
b (3–5 years) SM affected	41.18% (*n* = 17)	44.44% (*n* = 9)	50.00% (*n* = 12)	25.00% (*n* = 4)	0.00% (*n* = 2)	50.00% (*n* = 6)
c (1–3 years) SM affected	23.08% (*n* = 13)	40.00% (*n* = 5)	28.57% (*n* = 14)	42.86% (*n* = 7)	100.00% (*n* = 3)	57.14% (*n* = 14)

Each cell represents the percentage and number of affected offspring for that specific parent combination.

**Table 8:** Effect of parental SM status and age of MRI scan of both parents on SM affected status of offspring.

Logistic regression analysis of the 336 combinations				
	Univariable analysis		Multivariable model (model of best fit)	
	Odds Ratio (CI)	*p*-Value	Odds Ratio (CI)	*p*-Value
Sex of offspring female (ref = male)	1.34 (0.74–2.39)	0.33		
Age of offspring (in years)	1.48 (1.19–1.89)	0.0008	1.45 (1.15–1.85)	0.002
Parental status (ref = 0 affected parents) 1 affected parent 2 affected parents	1: 1.37 (0.82–2.3) 2: 3.35(1.74–6.52)	1: 0.22 2: 0.0003	1: 1.32 (0.79–2.23) 2: 3.08 (1.58–6.04)	1: 0.29 2: 0.0009
Combination of the Age-category from scan of the parents, aa is reference category	ab: 0.68 (0.24–2.2) ac: 0.73 (0.27–2.12) bb: 0.46 (0.1–1.85) bc: 0.98 (0.37–2.72) cc: 0.66 (0.25–1.8)	0.48 0.56 0.29 0.97 0.41		

## Discussion

This study describes the effect of using MRI to reduce the prevalence of syringomyelia in the CKCS over a time period of 23 years. When the breeders started scanning, they followed the recommendations published in 2007 (32). At that time, the dogs were divided into five possible grades. If a CKCS showed clinical signs or was below 2.5 years of age and affected with an FFC of over 2 mm, the advice was not to breed with that dog. When this scheme, was refined in 2012 after Knowler et al. (33), the three age groups were introduced as SM gets worse over time. A CKCS can be normal at the age of 1 year and affected at the age of 3 years (5, 14, 20, 21, 33). Hence, SM grading was introduced using three classifications. Using both age and the classification into the three groups (SM0, SM1, and SM2), breeders could choose several combinations. In this study, we followed the three age groups, but we defined affected CKCS with a visible FFC on the transverse slides regardless of the exact width of the cavity. In our dataset, all affected CKCS had at least an FFC of 0.5 mm, and again, we confirmed that the odds to be diagnosed with SM d with age. Moreover, although the advice is not to combine two young CKCS (age group c) in our dataset, this was still the most frequent combination, and the majority of CKCS scanned are the younger dogs. As SM progresses over time, it would be more logical to scan, especially the older CKCS and only combine CKCS older than 3 years of age. However, this study describes 23 years of MRI selection, and in the beginning, the breeders were allowed to use all types of age combinations.

However, regardless of this, our study demonstrates that if breeders use only unaffected older CKCS, the risk of getting affected offspring clearly decreases. Over time, the prevalence of SM dropped from 38% (period 2010–2014) to 27% (period 2015–2019). It is commendable to see this decrease in SM, but the effect is less than expected as in the second period, the number of younger CKCS used has increased significantly. The result could have been better if other choices had been made. It is possible that the number of affected CKCS decreased in the second period simply because breeders that have affected young CKCS may choose not to rescan their CKCS at a higher age. The number of ‘age group b’ CKCS increased in the second period compared with the first and the percentage of ‘age group a’ CKCS decreased in the second period. The effect is most likely minimal as these numbers were not statistically significantly different. It is important that breeders scan their breeding stock, even when they have passed the age of 5 years, as this information is vital to calculate reliable estimated breeding values (EBVs). EBVs have been calculated before, and in 2017, our group reported that the EBV using the BVA/KC scheme was lower compared with an EBV calculated on the FFC width (21). If all CKCS would also be scanned after reaching the age of 5 years, it would, most likely, improve the effectiveness of selection.

These findings are in line with the remarks made in 2007 that selection is possible (32), and according to the study by Knowler et al. (33), it is possible to reduce the percentage of affected CKCS. They demonstrated that if both parents were affected, 92% of the offspring would have the risk of being affected, and if one parent was affected, the risk for the CKCS would be 77%. However, in that study, a CKCS was seen as unaffected, even if it had an FFC between 0 and 2 mm, when the dog was at least 2.5 years of age (grade A). In this study, we have defined a CKCS as unaffected if there is no visible CCD as this is the normal phenotype for any type of dog. To reduce SM effectively, the most logical choice is to select only unaffected CKCS. However, in our dataset, 43.8% of bitches and 34.7% of sires used for breeding were affected with SM. Moreover, although the odds ratio for affected offspring is slightly higher for an affected bitch compared with an affected sire (Tables 5, 6), sex does not significantly influence the odds of being affected with SM.

The current BVA/KC scheme allows seven different combinations of two affected CKCS. For instance, a CKCS older than 3 years of age, with an FFC of 2 mm (grade SM2b/a), is allowed to be mated with an unaffected CKCS provided the dog that is older than 5 years of age (grade SM0a). Several other combinations such as these are possible (see Text footnote 1, respectively). A very positive result of this study is that only 15% of the combinations were between two affected CKCS. Most combinations (46%) were between two unaffected CKCS. Moreover, although it reduces the risk of getting affected offspring, there is still a possibility that some CKCS of free combinations get affected offspring (Table 7).

There are some limitations. First of all, we were not able to calculate the effect of the grandparents. Knowler et al. (33), using the old grading system, noted an effect of the grandparents. As SM is a heritable disease, it seems logical that a CKCS whose parents are unaffected will have a higher breeding value compared with one or even two affected parents. However, at this stage, the number of offspring with scanned great parents in our dataset is too small to allow a statistical analysis. Second, a small number of CKCS had only one scanned parent. These are the CKCS that originate from the period prior to 2011. As of 2012, all Dutch and, as of 2023, all Danish CKCS are mandatory screened by MRI before breeding. That this mandatory screening is useful and is demonstrated in this study. Third, some of the MRI scans were made with a low field scanner, and it is possible that a small FFC is missed. It is possible that some CKCS would have been classified as affected if the scan was made with a high field scanner. It is expected that in time, the use of high field scanners will become more admissible for veterinary clinics, which will improve the quality of our diagnosis.

In light of this study, we make four recommendations. Recommendation 1 is to refine the current BVA/KC scheme in such a manner that a CKCS with a visible FFC of ≥0.5 mm, regardless of its age, is observed as affected. Recommendation 2 is not to breed with such a dog. Recommendation 3 is to use only sires that are found to be unaffected after reaching the age of 3 years or older. Moreover, recommendation 4 is, if possible, to apply the same to the bitches as well, only using them after reaching the age of 3 years and found to be unaffected at that time. This would, most likely, improve the effectiveness of MRI-based selection, and we speculate that the prevalence of SM will drop remarkedly more and in a shorter time than observed in this study.

However, a preliminary evaluation of this measurement, on the current Dutch and Danish breeding stock, would mean that up to 40% of the currently used CKCS would have to be excluded from breeding (unpublished data). This may introduce a genetic bottleneck as several other disorders, mentioned in the introduction (3–12), and diseases such as pancreatitis (36), platelet problems, and mitral valve regurgitation (37), and, among others, keratoconjunctivitis (38) are present in the population as well. Selective breeding in this breed may have detrimental effects on the genetic diversity (39), and therefore, we recommend the development of sophisticated breeding software using algorithms to reduce this risk.

## Conclusion

By screening their CKCS with MRI, breeders in the Netherlands and Denmark reduced the prevalence of SM. The risk of getting affected offspring is clearly reduced if breeders would only use unaffected older CKCS. To reduce the prevalence of SM, we advise all CKCS breeders worldwide, to screen their CKCS with MRI at 3 years of age prior to breeding and exclude all CKCS with an FFC ≥0.5 mm from breeding. To avoid an increase in the other diseases that have been described in this breed, the development of sophisticated breeding software using algorithms is desired to reduce this risk.

## Data availability statement

The raw data supporting the conclusions of this article will be made available by the authors, without undue reservation.

## Ethics statement

The requirement of ethical approval was waived by Animal Welfare Body Utrecht, Utrecht University, www.ivd-utrecht.nl for the studies involving animals because this study was performed on MRI data submitted by the owner after obtaining an informed consent. MRI’s were made to screen the dogs prior to breeding. The studies were conducted in accordance with the local legislation and institutional requirements. Written informed consent was obtained from the owners for the participation of their animals in this study.

## Author contributions

CL: Data curation, Formal analysis, Methodology, Writing – original draft, Writing – review & editing. VS: Data curation, Writing – review & editing. HF: Data curation, Formal analysis, Methodology, Supervision, Writing – review & editing. PM: Conceptualization, Data curation, Formal analysis, Investigation, Methodology, Project administration, Resources, Supervision, Validation, Visualization, Writing – original draft, Writing – review & editing.

## Funding

The author(s) declare financial support was received for the research, authorship, and/or publication of this article. The publication of this manuscript is financially supported by the IVC Evidensia Research Fund.

## Footnotes

^1^See: British Veterinary Association (BVA)/Kennel Club (KC) scheme. www.bva.co.uk/Canine-Health-Schemes/CM-SM-Scheme (accessed 19 October 2023).

^2^ https://www.r-project.org/

^3^Excel Microsoft® 2023.
